# Findings from the Norwegian multicenter study of the DSM-5 alternative model for personality disorders: baseline and 8-year follow-up

**DOI:** 10.1192/j.eurpsy.2026.10315

**Published:** 2026-07-31

**Authors:** B. Hummelen, I. Eikenæs

**Affiliations:** Clinic Mental Health and Addiction, Oslo University Hospital, Oslo, Norway

## Abstract

**Abstract:**

Predictive validity is the sine qua non of any diagnostic system. Although prior research supports the clinical utility of the DSM-5 Alternative Model for Personality Disorders (AMPD), longitudinal interview-based studies examining whether impairment of personality functioning predicts long-term psychosocial and occupational outcomes remain limited.

The Norwegian Multisite Study of the AMPD (NorAMP) included 282 clinically assessed patients at baseline (2015–2016), of whom 153 participated in an 8-years follow-up study. Patients were recruited from a variety of clinical sites, including mental health outpatient departments, inpatient departments, addiction treatment units, and units specialized in the assessment and treatment of personality disorders. The latter units were all part of the Norwegian Network for Personality Disorders, a large clinical network including 18 treatment units.

Traditional personality disorder diagnoses were assessed using the Structured Clinical Interview for DSM-5 Personality Disorders (SCID-5-PD). The Level of Personality Functioning Scale (LPFS) was assessed using the Structured Clinical Interview for DSM-5 AMPD (SCID-5-AMPD-I), administered by trained clinicians at baseline and 8-years follow-up.

Preliminary longitudinal analyses indicate modest rank-order stability of global LPFS across eight years (r ≈ .37), suggesting both continuity and substantial individual change. Baseline LPFS severity was significantly associated with long-term psychosocial impairment and occupational functioning. More findings will be presented at the symposium.

**Disclosure of Interest:**

None Declared

